# Evaluation of DNA extraction yield from a chlorinated drinking water distribution system

**DOI:** 10.1371/journal.pone.0253799

**Published:** 2021-06-24

**Authors:** Ratna E. Putri, Lan Hee Kim, Nadia Farhat, Mashael Felemban, Pascal E. Saikaly, Johannes S. Vrouwenvelder

**Affiliations:** 1 Biological and Environmental Science and Engineering Division, Water Desalination and Reuse Center, King Abdullah University of Science and Technology, Thuwal, Saudi Arabia; 2 Faculty of Applied Sciences, Department of Biotechnology, Delft University of Technology, Delft, The Netherlands; Universita degli Studi di Milano-Bicocca, ITALY

## Abstract

Desalination technology based on Reverse Osmosis (RO) membrane filtration has been resorted to provide high-quality drinking water. RO produced drinking water is characterized by a low bacterial cell concentration. Monitoring microbial quality and ensuring membrane-treated water safety has taken advantage of the rapid development of DNA-based techniques. However, the DNA extraction process from RO-based drinking water samples needs to be evaluated regarding the biomass amount (filtration volume) and residual disinfectant such as chlorine, as it can affect the DNA yield. We assessed the DNA recovery applied in drinking water microbiome studies as a function of (i) different filtration volumes, (ii) presence and absence of residual chlorine, and (iii) the addition of a known *Escherichia coli* concentration into the (sterile and non-sterile, chlorinated and dechlorinated) tap water prior filtration, and directly onto the (0.2 μm pore size, 47 mm diameter) mixed ester cellulose membrane filters without and after tap water filtration. Our findings demonstrated that the co-occurrence of residual chlorine and low biomass/cell density water samples (RO-treated water with a total cell concentration ranging between 2.47 × 10^2^–1.5 × 10^3^ cells/mL) failed to provide sufficient DNA quantity (below the threshold concentration required for sequencing-based procedures) irrespective of filtration volumes used (4, 20, 40, 60 L) and even after performing dechlorination. After exposure to tap water containing residual chlorine (0.2 mg/L), we observed a significant reduction of *E*. *coli* cell concentration and the degradation of its DNA (DNA yield was below detection limit) at a lower disinfectant level compared to what was previously reported, indicating that free-living bacteria and their DNA present in the drinking water are subject to the same conditions. The membrane spiking experiment confirmed no significant impact from any potential inhibitors (e.g. organic/inorganic components) present in the drinking water matrix on DNA extraction yield. We found that very low DNA content is likely to be the norm in chlorinated drinking water that gives hindsight to its limitation in providing robust results for any downstream molecular analyses for microbiome surveys. We advise that measurement of DNA yield is a necessary first step in chlorinated drinking water distribution systems (DWDSs) before conducting any downstream omics analyses such as amplicon sequencing to avoid inaccurate interpretations of results based on very low DNA content. This study expands a substantial source of bias in using DNA-based methods for low biomass samples typical in chlorinated DWDSs. Suggestions are provided for DNA-based research in drinking water with residual disinfectant.

## Introduction

World Resources Institute reported that water withdrawal has doubled from the 1960s until 2019 due to the growing world’s economy and population [1]. The current water resources and conventional approaches (e.g., groundwater and surface water) are no longer sufficient to meet human needs, especially in water-stressed countries [1,2]. The capacity and application of seawater reverse osmosis (SWRO) desalination technology has been rapidly growing worldwide, and many countries within the Arabian Peninsula, North Africa, and South Asia have already resorted to seawater desalination to alleviate their water supply [3]. This technology seems to offer an unlimited, steady supply of high-quality water [4]. Among the seven current categories of desalination technology present [2], reverse osmosis is known to be the most effective membrane-based filtration method for drinking water production with the ability to remove almost 99% of the bacterial cells and more than 5 log removal values of viruses [5,6]. In addition, efforts have been made to decrease the unit water cost by enhancing RO process efficiency, improving operating conditions and applying hybrid system [7,8].

Once the drinking water is produced, ensuring the biological stability and monitoring the water quality during distribution to/and at the point-of-use is paramount [9,10]. RO permeate water usually undergo post-treatment process such as remineralization, aeration that influence the final (microbial) water quality [11]. Besides the conventional culture-dependent techniques, culture-independent based methods such as quantitative PCR (qPCR), DNA-based or RNA-based sequencing have been applied to detect and monitor drinking water quality indicators/opportunistic bacteria (e.g., Total and Fecal Coliforms, *Legionella pneumophila*). These techniques have increased the understanding of microbial ecology in DWDSs [12,13].

For characterization of drinking water microbial communities present in the bulk water, the water volumes filtered are usually between 0.05 to 5 L to collect cell biomass and isolate DNA for diversity estimates [14–16]. However, there is no consensus on the sampling volume required for optimal DNA recovery from drinking water [12,17].

Reverse osmosis (RO) treatment can produce drinking water with significantly low bacterial cell concentrations [5], and residual chlorine application can further reduce bacterial loads in the delivered drinking water [18,19]. At the microbial community levels, chlorine reduced the relative abundance of Proteobacteria phylotypes [20] and created a more homogenous bacterial population [21]; while at the molecular levels, a decrease in DNA concentrations has been observed with an increase of chlorine exposure time [22]. It is common to perform dechlorination in drinking water microbiome studies [23–26] although some studies have demonstrated the detrimental effect of chlorine on the bacterial cells and their DNA [27–29] that challenges the interpretation of DNA-based studies in chlorinated DWDSs. To the best of the authors’ knowledge, no evaluation study has been done addressing the effect of biomass amount (i.e., filtration volume) and residual chlorine on the DNA yield.

This study aims to investigate: (i) the impact of different filtration volumes, (ii) the effect of residual chlorine presence/absence, and (iii) the effect of spiking using *Escherichia coli* (into the water and onto the membrane filter) on the DNA recovery from tap (drinking) water. Batch experiments were designed in three scenarios: 1) tap water without *E*. *coli* spiking, 2) with *E*. *coli* spiking into the (sterile and non-sterile—indigenous microbes present/absent—and chlorinated/chlorine maintained and dechlorinated/chlorine removed) tap water, and 3) with *E*. *coli* spiking onto the membrane filter without and after tap water filtration. This study aids in understanding the challenges associated with DNA extraction from chlorinated drinking water distribution systems (DWDSs) that may suffer from bacterial cell destruction and DNA degradation. This paper provides additional insight into potential biases that may arise from using DNA-based methods for studying the microbiomes in DWDSs.

## Materials and methods

### Experimental design

**Fig 1:**
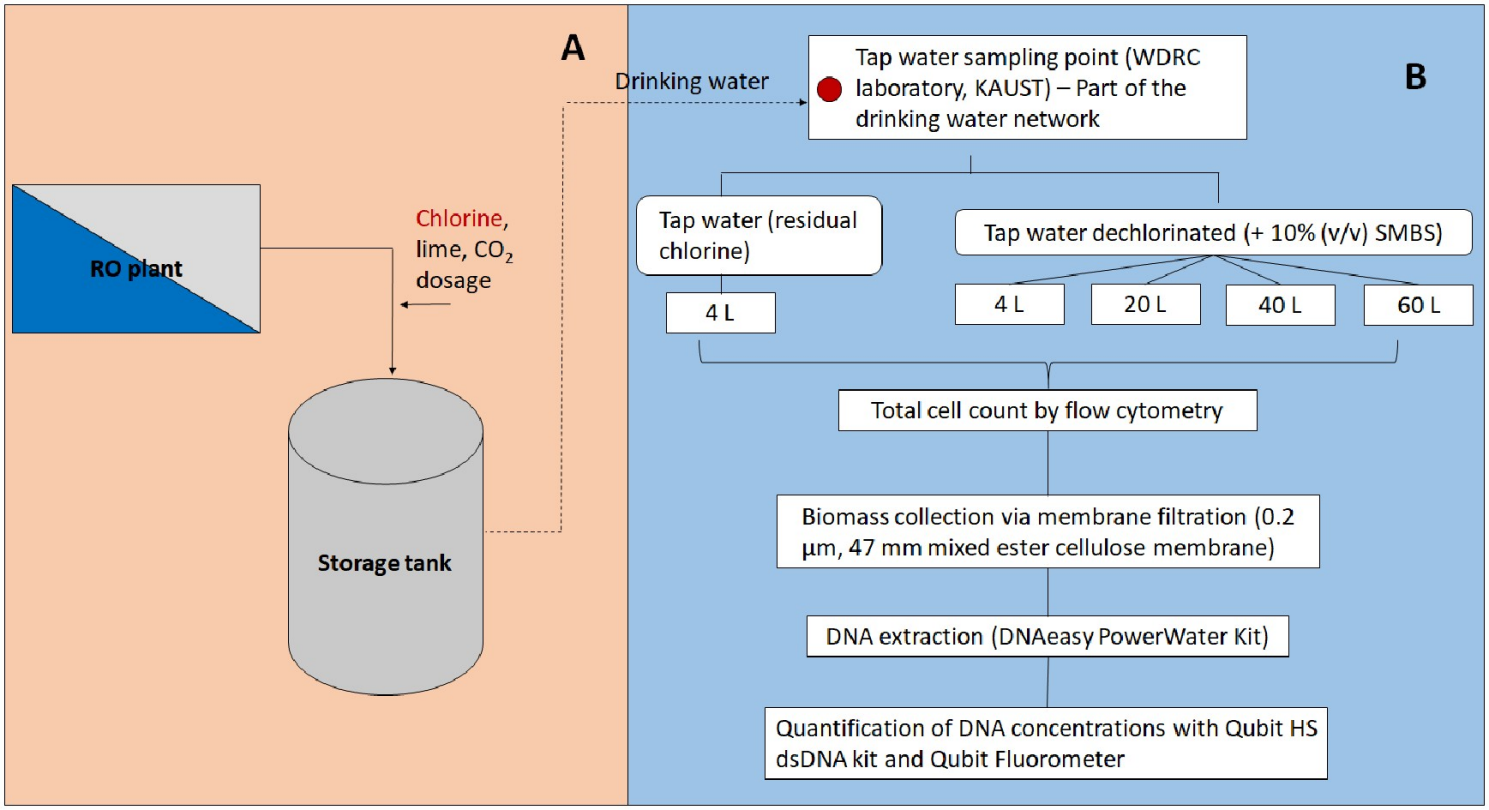
The upstream part of KAUST seawater reverse osmosis plant (A) and the schematic overview of the DNA recovery evaluation with different filtration volumes from tap (drinking) water in this study (B). A 10% (v/v) solution of sodium metabisulfite (SMBS) (Na_2_S_2_O_5_) was used to quench the free chlorine (referred as dechlorination).

To evaluate DNA extraction yield, (drinking) tap water was collected in triplicate for different filtration volumes (4, 20, 40, 60 L) from a single (drinking) tap in the Water Desalination and Reuse Center (WDRC) laboratory, King Abdullah University of Science and Technology, Saudi Arabia (Fig 1B). The tap water is produced by seawater reverse osmosis (SWRO) membrane systems in KAUST, and chlorine is added to maintain a residual disinfectant during distribution. The KAUST SWRO process description has been mentioned in previous studies [30,31]. Before the sampling, the tap was opened for 2–5 minutes at a moderate flow rate to achieve network quality water. Polystyrene bottles (Corning^®^, USA) that were previously sterilized using 0.5% (v/v) of sodium hypochlorite (NaOCl), followed by thorough rinsing with deionized (DI) water were used for tap water sample collection.

### Preparation of (sterile, non-sterile, chlorinated, dechlorinated) water samples

**Fig 2:**
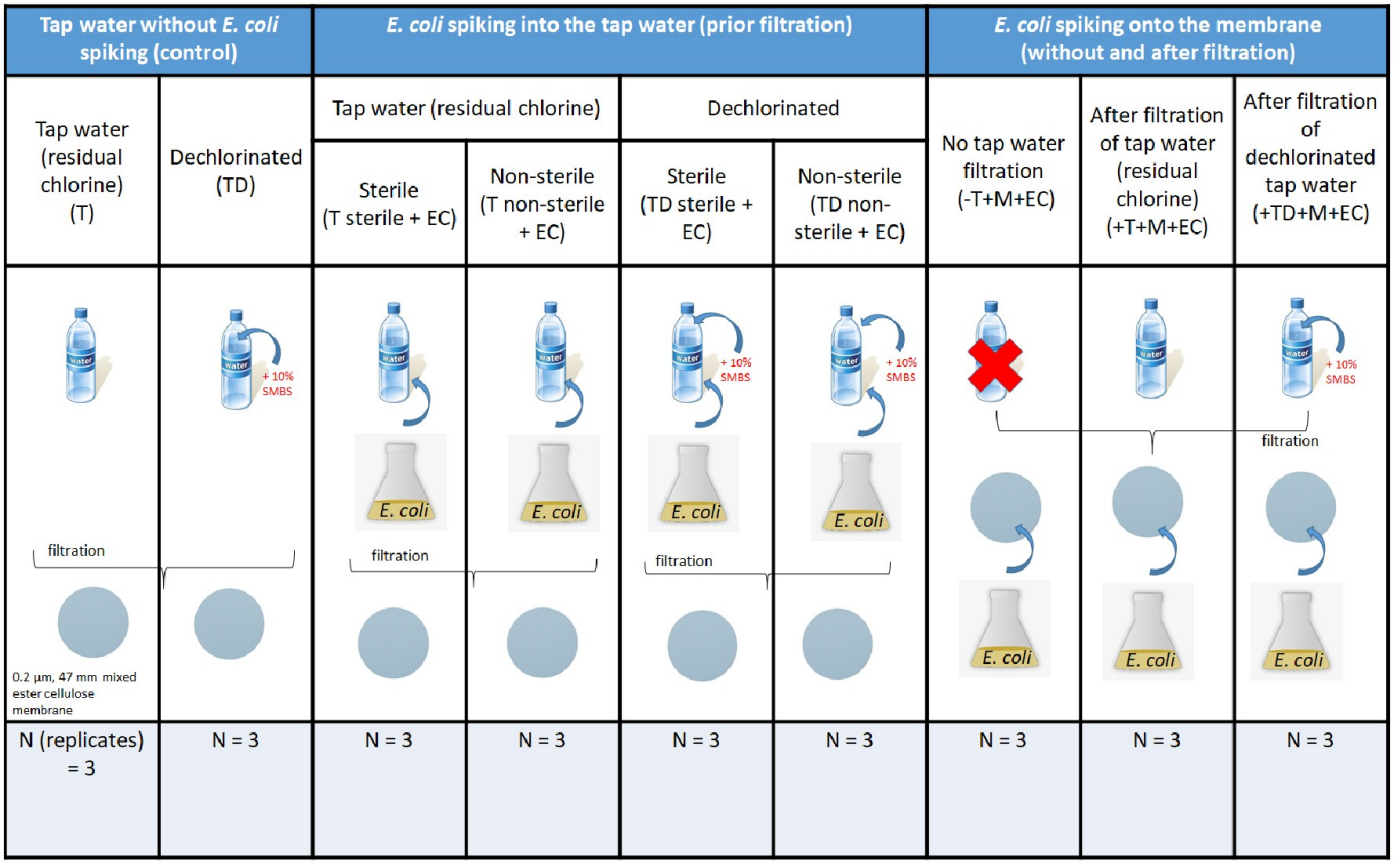
Scheme of the batch experiments for evaluating DNA extraction yield via addition of *E*. *coli* cell solution. The experiments were designed with three main scenarios: Tap water without *E*. *coli* spiking, tap water with *E*. *coli* spiking prior filtration, membrane spiking with *E*. *coli* without and after tap water filtration; and sub-scenarios (T: Tap water with residual chlorine, TD: Dechlorinated tap water, sterile: Tap water was pre-sterilized with membrane filtration to remove indigenous microbes, non-sterile: Tap water with no prior pre-filtration). Each replicate (n) comprises of four liters of tap water. Each treatment is explicitly assigned with the code shown at the second row of the table. Sterile mixed ester cellulose membrane filter (0.2 μm, 47 mm diameter) was used for tap water filtration (i.e., biomass collection). A 100 μL of 10% (v/v) solution sodium metabisulfite (SMBS) (Na_2_S_2_O_5_) was used as the dechlorinating agent.

Batch experiments were designed to assess the effect of residual chlorine and addition of *Escherichia* coli on DNA yield. The batch experiments evaluated (i) the presence and removal of chlorine; and (ii) addition of known concentration of *E*. *coli* into tap water (sterile and non-sterile; presence and absence of residual chlorine), and onto the membrane filter (with and without filtration of tap water) (Fig 2).

Tap water (4 L) containing residual chlorine (referred to as chlorinated) was taken from a single tap in our laboratory on the same day in sterile polystyrene bottles (Corning^®^, USA). Tap water was quenched (referred to as dechlorinated) by adding a 10% (v/v) sodium metabisulfite or SMBS (Na_2_S_2_O_5_) of pH 7.6 [32]. Theoretically, each part of chlorine residual requires 1.34 parts of SMBS (i.e., 1.34 mg/L SMBS to neutralize 1 mg/L of chlorine) [32]. In practice, 50 μL was effective to dechlorinated one liter of tap water containing 0.2 mg/L residual chlorine but particularly in this study, the dosage was doubled to diminish the minute effect of chlorine. KAUST tap water have pH around 8.9–9.2 and addition of 10% (v/v) SMBS solution reduced pH, thus adjustment was made using NaOH or HCL 0.1 N to ensure that the batch experiment was subjected to the same conditions (see S1 Table in Supporting Information). For the *E*. *coli* spiking experiment, pH of tap water was set to 7.6 in order not exposing bacteria to harsh conditions.

To prepare sterile tap water, the tap water (4 L) was pre-filtered through a 0.2 μm, 47 diameter of sterile mixed ester cellulose membrane filter (Millipore, USA) to remove indigenous microbial community. Meanwhile, non-sterile (either chlorine maintained or removed) tap water received no prior sterilization.

To check the physicochemical properties of the tap water, pH was measured using a multimeter probe (WTW, Germany), while total chlorine in the water samples was determined by the diethyl-p-phenylenediamine (DPD) method using Hach Pocket Colorimeter^™^ II. Water properties are presented in S1 Table (Supporting Information).

### Preparation of *E*. *coli* cell stock solution

*Escherichia coli* (DSM 1103) was first incubated in Luria Bertani (LB) broth (BD, USA) overnight at 37°C and 120 rpm. The cultured *E*. *coli* were sub-cultured twice in LB broth to maintain bacterial activity before use. The bacterial cells were harvested by centrifugation at 6,953×g (~8000 rpm) for 10 min, and the pellet was washed twice with sterilized 1× phosphate-buffered saline (PBS) solution. The harvested *E*. *coli* pellet was then suspended in a 50 mL sterile PBS (referred to as the stock solution of *E*. *coli*).

### Quantification of bacterial cell concentration using flow cytometry

The concentration of *E*. *coli* cell in the stock solution was determined before spiking into the water and onto the membranes using an Accuri C6 Plus flow cytometer (BD Biosciences, USA). Determination of total bacterial cell concentration using flow cytometry (FCM) and staining procedures was done as described in previous studies [33–35]. In brief, 990 μL of water sample was pipetted into a 2 mL sterile amber tube, stained with 10 μL of SYBR Green I (100× diluted from stock in sterile DI water), and incubated at 37°C for 10 minutes. A 200 μL of the stained samples were later transferred to a 96-well plate, and the bacterial cell concentration was measured. Cell counting was carried out by enumerating the number of events in a 50 μL volume (default setting) that pass through the fluidics system. A fixed gate was set on FL1 > 600 at a fast flow rate of 66 μL/min. Electronic gating on density plots was set on green versus red fluorescence (FL1: 533/30 nm/FL3: > 670 nm). The lower detection limit for total cell quantification was 1000 cells/mL (see S3 Fig and *Detection limit of flow cytometry used in this study*-*methods* in Supporting Information), similar to previous report [36]. To perform batch experiments, the concentration of *E*. *coli* used for spiking was adjusted to reach ~10^5^ cells/mL (water spiking) and ~10^7^ cells/cm^2^ (membrane spiking).

### DNA extraction

To collect the cell biomass, tap water was filtered with a 0.2 μm, 47 mm diameter of sterile mixed-estercellulose membrane (Millipore, USA). The addition of 100 μL of 10% (v/v) SMBS solution was done for dechlorinated sample designees and three hours of contact time before filtration was maintained for all treatments to minimize the effect of a minute amount of chlorine present after quenching. For the spiking experiment, *E*. *coli* (to reach a final of concentration 10^5^ cells/mL in 4 L tap water) was added, and the same three hours of contact time were used to attenuate the *E*. *coli* before filtration (i.e., biomass collection). For the membrane spiking, *E*. *coli* was directly spiked (spiking concentration 10^7^ cells/cm^2^) onto the membrane filter, and after tap water (chlorinated/dechlorinated) filtration. All membrane filters containing cell biomass were stored at -20°C until DNA extraction was performed.

The DNA was extracted using a DNeasy PowerWater Kit (Qiagen, Germany) according to the manufacturer’s instructions. The kit yields higher DNA amount and has a better reproducibility and a broad OTUs identification fits for drinking water microbiome study [14]. The DNA extraction was performed in three independent replicates and a negative control (sterile/virgin membrane) was added to quantify possible contamination from the materials used, or the environment (e.g., sample handling, extraction kit, membrane and air contact). The quantification of DNA concentration was performed using Qubit^®^ 4 Fluorometer (Thermo Fisher Scientific, USA) and Qubit dsDNA High Sensitivity (HS) Assay Kit (detection range 0.01–100 ng/μL) (Thermo Fisher Scientific, USA).

### Statistical analysis

All plots were generated using OriginPro (version 2019). All statistical analyses, including descriptive statistics and regression were performed with R studio version 3.6.1 [37]. One-way ANOVA for comparison of means and post-hoc Tukey’s test for multiple pairwise comparisons of sample groups was used to determine significant differences in DNA concentrations between and within treatment groups/sub-treatment groups (Fig 2). Correction for multiple inferences was done using Benjamini-Hochberg methods by adjusting *p* values to ensure false discovery rate upper bound. The level of significance was set at *p* <0.05.

## Results

### DNA extraction yield of tap water with different filtration volumes

**Fig 3:**
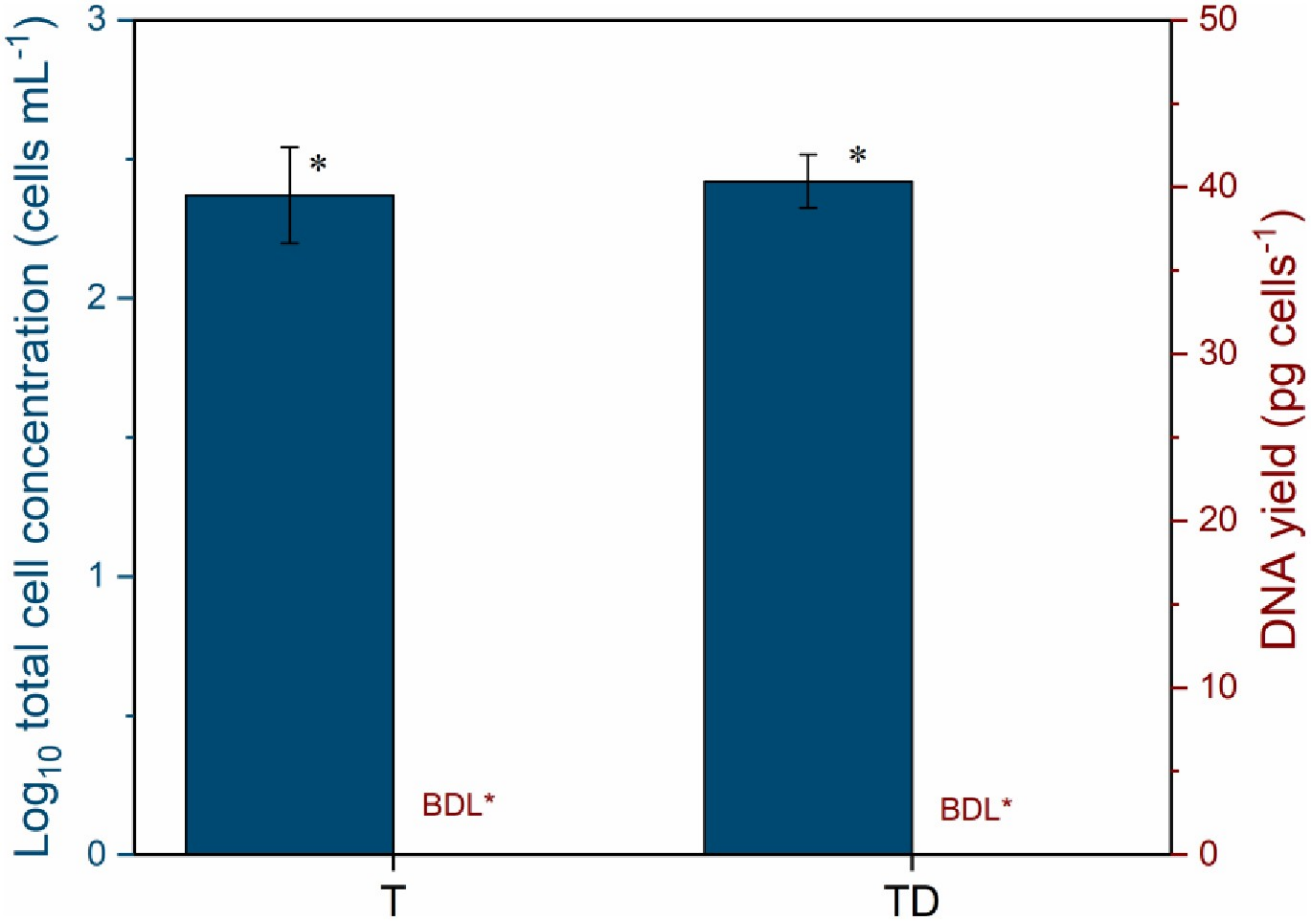
Cell concentration and DNA yield obtained from (T) tap water with residual chlorine and (TD) dechlorinated tap water. The tap (drinking) water treatments were done by maintaining or removing residual chlorine, as summarized in Fig 2. A blue-coloured star indicates that total bacterial cell concentration was below the limit of detection (LOD) of flow cytometry which is 10^3^ cells/mL. A red-coloured star indicates that the DNA concentration measured was below detection limit (BDL) of Qubit High Sensitivity assay dsDNA ≤ 0.01 ng/μL. The error bars show errors from three independent replicates.

Evaluation of the DNA recovery with varying filtration volumes of tap water (details are presented in Figs 1 and 2) with three independent replicates, whether it maintained residual chlorine (T: chlorinated) or not (TD: dechlorinated), showed low DNA yields. None of the samples had measurable DNA concentrations (limit of quantification of Qubit HS dsDNA assay is 0.01 ng/μL). There was no difference in the bacterial cell concentration between dechlorinated tap water and the tap water with residual chlorine (T and TD, Fig 3). Tap water had a low total cell concentration of two-log_10_ cells/mL (below the limit of detection of FCM at 10^3^ cells/mL, S3 Fig) even after the removal of chlorine (Fig 3). Fig 3 also indicates that dechlorination did not improve DNA yield.

Different sampling volumes (4, 20, 40, 60 L) of tap water were filtered (summarized in Fig 1), anticipating higher cell biomass and DNA yield. On the contrary, higher volumes did not correspond to a proportional increase in the DNA concentrations. The amount of DNA was below the detection limit in all samples (Table 1). A subsequent PCR also resulted in similar DNA concentrations around 6 ng/μL (Table 1). Overall, irrespective of filtration volumes and dechlorination, there was no impact proportional to the DNA yield. As visualized by qualitative gel electrophoresis, the absence of intact DNA signifies the damaging effect of chlorine on the DNA (S1 Fig).

**Table 1:** DNA recovery from different filtration volumes of dechlorinated tap water before and after PCR, means ± SD.

Sample volume (L)	Extracted DNA concentration (ng/μL)	PCR concentration^b^ (ng/μL)
4	≤ 0.01^a^	6.41 ± 0.70
20	≤ 0.01	6.22 ± 0.62
40	≤ 0.01	5.75 ± 0.60
60	≤ 0.01	6.09 ± 0.34

^a^Below detection limit of Qubit HS dsDNA assay.

^b^PCR concentration is the concentration of amplicon PCR based on 16s rRNA gene (see Supporting Information S3 Text for *PCR amplification of extracted DNA-methods*). Measurement of DNA and PCR concentration was done with Qubit dsDNA HS assay.

### DNA extraction yield of tap water spiked with *E*. *coli*

The spiking experiment was performed to compare the DNA yield between the original and spiked cell concentrations, both for chlorinated and dechlorinated tap water. Details of the spiking are presented in S2 Table (Supporting Information). The results revealed that DNA yields differ between spiked and non-spiked drinking water samples that were co-influenced by the presence of residual chlorine.

**Fig 4:**
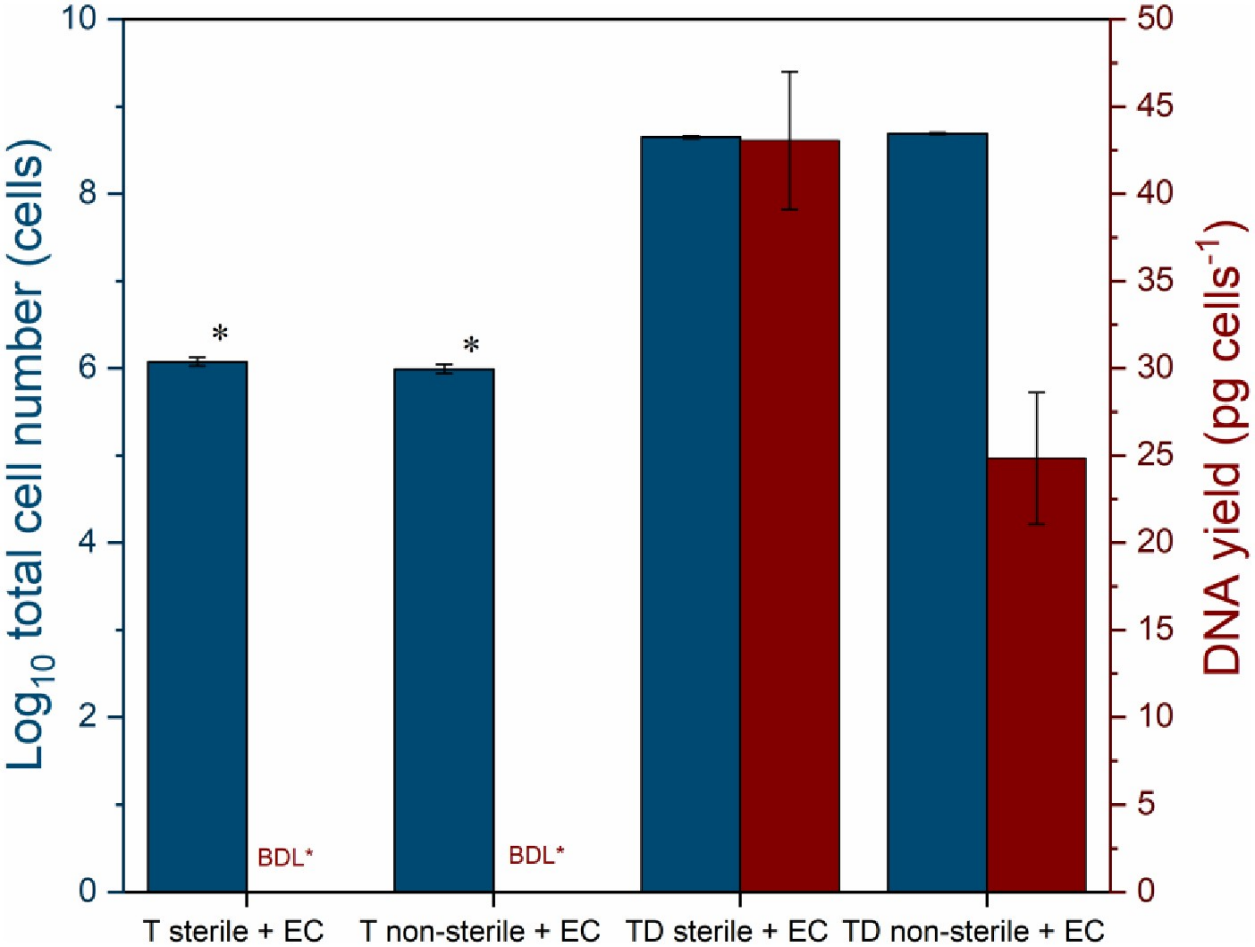
Total cell numbers and DNA yield obtained after *Escherichia coli* spiking into sterile and non-sterile (T) tap water with residual chlorine and (TD) dechlorinated tap water prior membrane filtration. The details for (sterile, non-sterile) tap water treatments used for spiking treatments are summarized in Fig 2. Sterile: Tap water was pre-filtered by membrane filtration (0.2 μm, 47 mm diameter) before spiking. Non-sterile: Tap water without sterilization. + EC: Addition of *Escherichia coli* solution. A blue-coloured star indicates that total bacterial cell concentration was below the limit of detection (LOD) of flow cytometry which is 10^3^ cells/mL. A red-coloured star indicates that the DNA concentration measured was below detection limit (BDL) of Qubit High Sensitivity assay dsDNA ≤ 0.01 ng/μL. The error bars show errors from triplicate samples.

The impact of indigenous microbes’ presence in tap water was also assessed via pre-sequential membrane filtration of tap water before spiking. *E*. *coli* cells were maintained in dechlorinated tap water, i.e., cell recovery was within the same order of magnitude of expected final total cell concentration (around 96% cell recovery, S2 Table in Supporting Information). The total cell numbers, however, substantially lower than the detection limit of FCM (0.2% cell recovery) in tap water that contained residual chlorine (0.2 mg/L) with *E*. *coli* spiking within three hours of contact time (Fig 4 and S2 Table in Supporting Information).

With the absence of the spiked *E*. *coli* cells in the chlorinated tap water, the DNA yield was below the detection limit, both for (T sterile + EC) sterile and (T non-sterile + EC) non-sterile tap water (Fig 4). In contrast, a considerably higher DNA yield was obtained from spiked dechlorinated tap water (TD sterile/non-sterile + EC) proportional to the increase of total cell number (Fig 4). To sum up, the addition of *E*. *coli* biomass and removal of residual chlorine substantially increased DNA yield. This study confirmed DNA degradation at a lower chlorine concentration than what was previously reported in the literature. Additionally, note that spiked dechlorinated tap water (TD non-sterile + EC, containing indigenous microbes) had a 40% reduction of DNA yield compared to sterile dechlorinated tap water spiked with *E*. *coli* (TD sterile + EC).

### DNA yield of membrane filters spiked with *E*. *coli*

**Fig 5:**
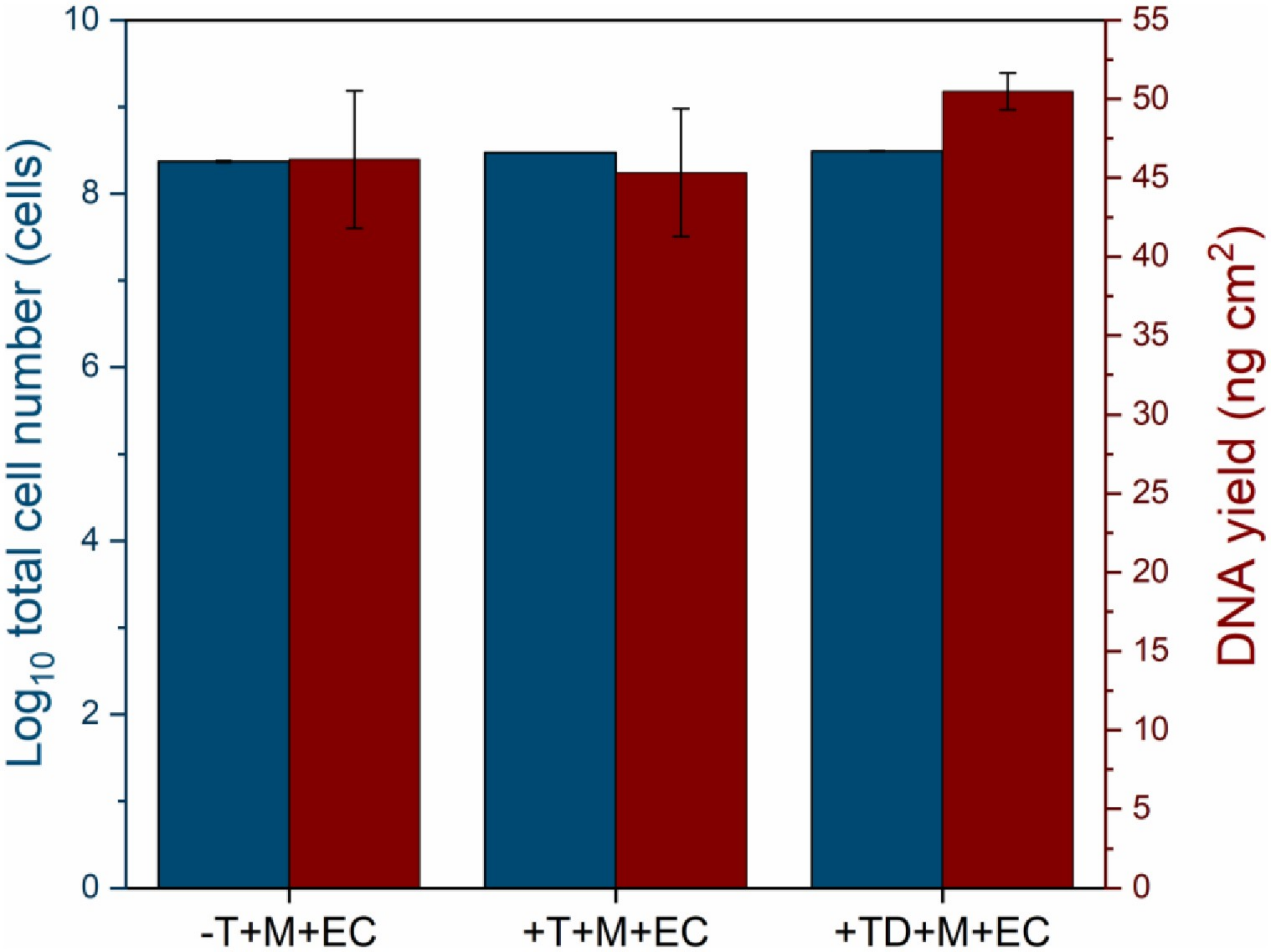
Total cells and DNA yield obtained from spiking onto membrane filters without and after tap water (T: Tap water with residual chlorine and TD: Dechlorinated tap water) filtration. The details for membrane spiking treatments are summarized in Fig 2. T: Tap water containing residual chlorine. TD: Tap water with the removal of residual chlorine (addition of SMBS 10% (v/v)).–T + M + EC: Membrane spiked with *E*. *coli* only (without tap water filtration). +T + M + EC: Membrane spiked with *E*. *coli* after tap water (with residual chlorine) filtration. +TD + M + EC: Membrane spiked with *E*. *coli* after tap water (dechlorinated) filtration. The error bars show errors from triplicate samples.

To assess the impact of the RO water matrix (i.e., organic/non-organic constituents) retained on the membrane filter towards DNA recovery, *E*. *coli* was spiked directly onto the membrane. *E*. *coli* was injected onto the membrane filters (details in S2 Table Supporting Information) after filtration of chlorinated (T) and dechlorinated tap water (TD), and also a negative control (-T: without tap water filtration, Fig 5). Similar total cell numbers were obtained from all treatments (10^7^ cells/cm^2^, S2 Table). DNA yields from all the spiked membrane treatments had no comparable differences, consistent with the total cell numbers (Fig 5). Apparently, the accumulated components (i.e., organic/inorganic compounds) present in the water (S2A Fig in Supporting Information) did not affect DNA recovery from the membrane filters.

**Fig 6:**
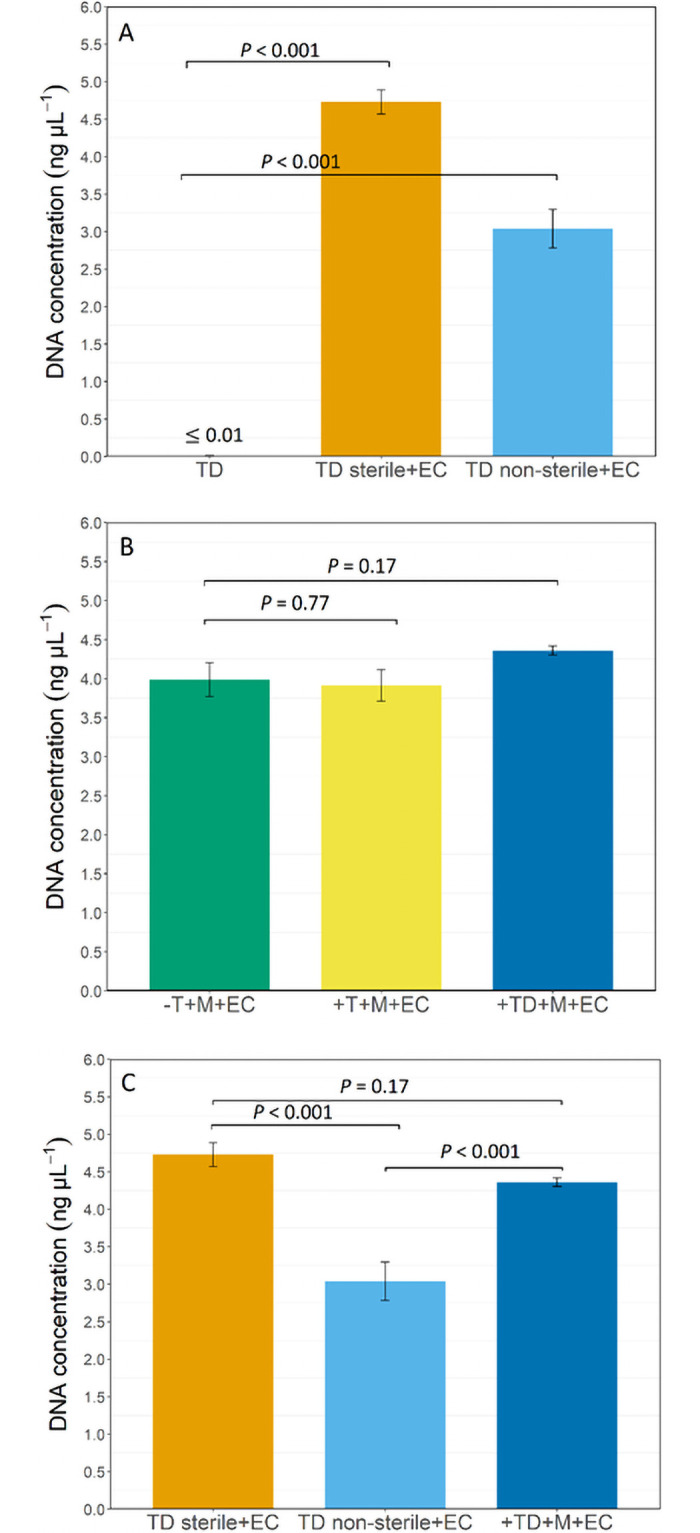
The extracted DNA concentration collected from dechlorinated tap water (A), membrane filters (B) spiked with *Escherichia coli*, and its subset (C) (sterile, non-sterile) dechlorinated tap water and membrane spiked with *E*. *coli* after filtration of dechlorinated tap water). + EC: Addition of *Escherichia coli*. TD: Dechlorinated tap water (addition of SMBS 10%). TD sterile: Dechlorinated tap water received prior membrane sterilization. TD non-sterile: Dechlorinated tap water without prior tap water sterilization.–T + M + EC: Membrane only (without tap water filtration) with the addition of *E*. *coli*. +T + M + EC: Membrane spiked with *E*. *coli* after filtration of tap water (residual chlorine). +TD + M + EC: Membrane spiked with *E*. *coli* after filtration of dechlorinated tap water. The error bars show errors from triplicate samples. *P*-value was shown for pairwise comparison (vs. control designees) based on ANOVA and post hoc Tukey’s test.

None of the chlorinated tap water (T) samples were included in the statistical test (One-way ANOVA) due to the insufficient DNA amounts (i.e., below detectable level ≤ 0.01 ng/μL). For the (TD) dechlorinated tap water, DNA concentrations were significantly higher after *E*. *coli* spiking, whether the water was sterilized or not, compared to the control (TD: dechlorinated tap water without *E*. *coli* spiking) (*p* < 0.001, Fig 6A). The similar DNA concentration obtained from the spiked membrane treatments demonstrate the negligible impact from tap water constituents (i.e. organics and inorganics as summarized in S3 Table and S4 Fig in Supporting Information) retained on the membrane filters towards the DNA extraction efficiency (*p* > 0.05, Fig 6B). There was no substantial difference for the DNA amount yielded from sterile dechlorinated water (TD sterile + EC) spiked with *E*. *coli* prior filtration compared to the spiked membrane filter (+TD + M + EC) after filtration (*p* > 0.05, Fig 6C). However, the DNA concentration reduced significantly for the non-sterile dechlorinated tap water (TD non-sterile + EC) compared to both sterile dechlorinated tap water (TD sterile + EC) and membrane spiked with *E*. *coli* after dechlorinated tap water filtration (+TD + M + EC; *p* < 0.001, Fig 6C). In summary, the spiked membrane spiked experiment shows that constituents (originated from the tap water matrix) accumulated on the membrane filters during filtration had no pronounced impact on the DNA extraction yield.

## Discussion

Batch experiments with three independent replicates were used to evaluate the DNA extraction yield from a chlorinated drinking water system based on: (i) different filtration volumes, (ii) presence and removal of chlorine, and (iii) spiking treatments of *Escherichia coli* (into the water suspension and onto the membrane filters) (details in Figs 1 and 2). To our knowledge, no study has evaluated the accuracy of the low quantity (or absence) of DNA related to residual chlorine presence on the interpretation of drinking water microbiome study. By performing spiking experiments using *E*. *coli*, we elucidated the effect of residual chlorine, RO-treated drinking water matrix on the recovery of cell biomass, and the impact of indigenous (background) microbes towards DNA recovery.

### No impact of water volume on DNA recovery

In this study, the RO-treated tap (drinking) water samples that maintained residual chlorine harbored total bacterial cell concentration between 2.47 × 10^2^–1.5 × 10^3^ cells/mL, in agreement with previously reported studies [38–40]. Irrespective of filtration volumes (4, 20, 40, 60 L), the yield of DNA was very low (i.e., ≤ 0.01 ng/μL, below detection limit, Table 1). This result coincides with the previous report that found insufficient DNA quantity (sample volume filtered was 100 L of microfiltration-reverse osmosis (MF-RO) treated water receiving post-treatment ultraviolet advanced oxidation process) [38]. The same author reported that the result of 16s rRNA qPCR from those samples was below the negative control (< 10^2^ copies genes/mL) and thus prevented further sequencing analysis (i.e., metagenomics and metatranscriptomics) [38]. Our study confirms a vast biomass reduction in the drinking water produced by RO, as previously reported [39–41]. The presence of residual chlorine further suppresses the bacterial content (i.e., lower cell density) in the water.

The required sampling volume for different environments (i.e., drinking water sources/treatments) will differ provided that it gives a representative sample diversity [42,43]. For drinking water microbiome studies, different sampling volumes in the range of 0.05–5 L were reported for DNA extraction [14]. In the current study, increasing the water filtration volume from 4L to 60L did not result in increased DNA recovery, which is in contrast to the suggestion made by several studies [15,44].

Aiming to increase DNA yield by increasing the biomass amount is problematic because the mean DNA yield per cell has been reported to have a negative correlation with cell density (as the total bacterial cell count increases, the DNA yield per cell decreases) [45,46]. Thus, normalizing the DNA recovery based on the mean mass of DNA per cell was proposed [46]. However, the mean mass will change according to the physiological factors (e.g., cell state, cell size, intra- and inter-variation of the bacterial genome within the microbial community) and physicochemical conditions of the environments [46–50].

In our case, the low DNA yield is very likely to be caused by extensive biomass reduction by the RO membrane (i.e., a larger fraction of RO-treated water would contain no cells) and the post-disinfection applied in the networks. The SEM images (S2A Fig) pinpoint the low presence (qualitative, no microscopy cell counting conducted; the total cell concentration was measured by flow cytometry in this study) of bacterial cells on the membrane filters, supporting this argument. The presence of chlorine is known to negatively affect planktonic bacteria’s growth in DWDSs [18,51]. In terms of the quality of extracted DNA, gel electrophoresis (S1 Fig) shows the prominent absence of intact DNA, indicating low DNA integrity caused by degradation [52,53].

The inherently low DNA yield in chlorinated drinking water samples has implications for the downstream analysis (sequencing-based procedures). Illumina recommends microbial genomic DNA concentration to be at least 5 ng/μL [54], and working at a lower boundary of this concentration needs to ensure enough replicates/sampling size and inclusion of negative controls (to identify potential cross-contamination) [55]. A rarefaction curve is also useful to assess whether the maximum sample diversity has been achieved and down amplification to lower reads (e.g., 10–100 reads/gene copies) is justifiable. However, low DNA concentration or any genetic markers have been demonstrated to give high false-negative results, increased false discovery and artefactual results [44,52] that is likely to be the case for sequencing-based studies in chlorinated drinking water [29]. We found that many reported drinking water microbiome studies from chlorinated systems (including 16s rRNA amplicon sequencing/metabarcoding, metagenomics) [20,56–64] often exclude raw experimental data of extracted DNA concentrations that is crucial to assess the quality of the sequencing results.

### Residual chlorine reduced the DNA yield of tap water and *E*. *coli* spiking

The total cell number after *E*. *coli* spiking was highly reduced in tap water containing residual chlorine due to the strong oxidant activity of the free chlorine species that damage bacterial cell membranes and oxidize their intracellular machinery (i.e., enzymes) [65,66]. The loss of cell membrane integrity of *E*. *coli* at < 1 mg/L of chemically-dosed chlorine (10 and 40 min contact time) [67], and reduction of intact cell concentration after chlorine exposure at 0.3–0.6 mg/L (up to 72 hours contact time) [68] have been reported. Following reduced *E*. *coli* cell concentration in tap water containing residual chlorine (0.2 mg/L), the subsequent DNA yield was below the detectable level (Fig 4). Our results negate the previous study that found no linear correlation between the significant reduction of the number of target bacterial cells with their genome quantity (i.e., DNA concentration) [69]. The present results are in agreement with a study that demonstrated (extracellular) DNA fragmentation and destruction of DNA carrying antibiotic resistance genes’ integrity caused by typical chlorine dosages (1–20 mg Cl_2_/L) [27]. The current results affirm DNA degradation at a lower level of chlorine (~0.2 mg/L) than previously reported [70]. DNA lesion (i.e., the kink of double-stranded DNA similar to pyrimidine dimers formation) is known to be the mechanism of the DNA degradation by chlorine [22,71].

### The RO water matrix had no impact on the DNA yield

The results show that water constituents (S3 Table in Supporting Information) retained on the membrane filters had no pronounced impact on the DNA yields from membrane spiked with *E*. *coli* treatments (Figs 5 and 6B). Organic materials and other biological particulates accumulating on the membrane filter has been known to affect DNA extraction efficiency [15]. Additionally, SEM images reveal deposits of water constituents on the membrane filters used for biomass filtration (S2A and S2B Fig in Supporting Information). In this study, however, no adverse inhibitory effect on DNA extraction yield was seen as there were no comparable differences between extracted DNA concentrations obtained from the membrane filters spiked with *E*. *coli* after tap water (with or without residual chlorine) filtration versus control (virgin membrane filters spiked with *E*. *coli* without tap water filtration, Fig 6B). While surface water used as RO feed water is known to contain humic-like substances that can inhibit the DNA extraction process [72], RO permeate has been reported to be deficient from humic-like substances that may cause DNA extraction failure [39]. The three dimensional spectra of tap water produced by RO membrane treatment used in this study confirms the absence of humic-like substances, i.e., a fluorophore signature at 300-340/400-450 nm (excitation/emission) that may inhibit DNA extraction process (S4 Fig).

In search of a possible explanation for the effect of indigenous microbes on reduction of DNA yield in non-sterile dechlorinated tap water spiked with *E*. *coli* (TD non-sterile + EC, Fig 4); a hypothesis could be the antagonistic effect of the indigenous microbial community towards *E*. *coli*. Vital and colleagues reported that *E*. *coli*:O157 growth was drastically restricted in the presence of competing bacteria in drinking water containing low nutrient concentration due to its inferior kinetic properties [73]. The origins of these microbial community may come from RO membrane elements and its O-ring seals seeding the network [74,75], or dislodged and survived planktonic cells from biofilm in occurrence with residual chlorine decay [51,76].

Another explanation for the resistance of *E*. *coli* in the presence of competing indigenous microbes is by forming aggregates and adhering to the batch container surfaces, i.e. biofilm formation [77]. The link between reduced DNA yields and biofilm or aggregate formation could be through *E*. *coli* extracellular DNA (eDNA) release. *E*. *coli* cells have been known to release eDNA through secretion in a planktonic state during static growth [78]. Depending on the strain, besides the structural role of eDNA for ’normal’ biofilm formation, eDNA can act as an adhering compound for cell-to-cell attachment in planktonic cultures [79,80]. eDNA also facilitates favorable acid-base interactions, explaining the effect of eDNA on aggregation and adhesion to the surface [81]. Our data cannot reveal the underlying mechanisms of the antagonistic effect and the presented hypotheses bases on the previous findings that can be considered for future studies. Nevertheless, the presence of indigenous microbes in dechlorinated RO-treated tap water had a significant impact towards *E*. *coli* and the obtained DNA yields.

A follow-up experiment could be performed to determine the time effect (incubation) following dechlorination (e.g., growth potential assay), and the effect of mixing ultra-pure quality water (membrane-treated) with drinking water treated with different processes (e.g., slow sand filtration, advanced water purification) or taken from different sources (e.g., surface, groundwater) which can contain higher microbial load on the final microbial water quality of the mixed water that are linked to a different functional (microbial) diversity [38].

### Implications

This study focusses on the poorly understood features [82–84] regarding the microbial DNA in chlorinated drinking water rather than focusing on evaluating extraction methods (or DNA extraction kits) to increase the DNA yield, which has been addressed elsewhere [14,85]. The present results reveal that low DNA quantity is likely to be the norm in chlorinated drinking water. The low DNA quantity may be a true reflection of low cell biomass/low cell density that could not be improved by increasing filtration volumes as presented in this study (Table 1). An attempt to use different DNA extraction methods like phenol-chloroform to increase DNA yield has its own inherent bias regarding the need for extensive purification, leading to a decreased DNA concentration, species diversity alteration, and low DNA purity [48,55,86].

The presence of low DNA quantity—as a consequence of low biomass/low cell density samples—in drinking water networks containing a residual disinfectant (e.g., chlorine) challenges the interpretation of DNA sequencing (e.g., 16S rRNA amplicon sequencing, metagenomics) data. Chlorinated drinking water samples have a total cell concentration between 103–10^5^ cells per/mL [18,87,88], and could be below 10^3^ cells/mL if the drinking water is produced by membrane-based processes like RO, as shown in this study. Analyses of microbial community composition of “low”-density samples (< 10^6^ cells per/mL) using 16S amplicon sequencing have shown changes in the relative abundance and representation of species, and overrepresentation of taxa in the phylum Proteobacteria [89]. Contamination from chemicals, kits and solutions [90,91] and the errors in sequencing are likely to introduce more taxa, causing shifts in the microbial profiles from the true profiles [89,90]. Therefore, shifts in “within-sample” diversity (alpha diversity) cannot be ruled out at a very low DNA concentration [89]. Further, potential error of using too low DNA concentration cannot be estimated or corrected for rare taxa and thus should be avoided [49]. However, we expect the impact of low DNA concentrations to be less pronounced on beta diversity (i.e., comparing bacterial communities between different samples) than alpha diversity. Nevertheless, we advise that it is imperative to report the concentration of extracted DNA to avoid biases in the interpretation sequencing data.

While using DNA-/RNA-based sequencing technologies (i.e., 454 pyrosequencing, Illumina, PacBio) have revealed more microbial functions than what previously known in different drinking water networks [92,93], understanding its limitations (i.e., the concordance of low quantity of the extracted microbial DNA) in chlorinated drinking water system is critical. We suggest that microbial community composition studies in chlorinated drinking water should integrate absolute cell quantification like flow cytometry (i.e., total and/or viable cell concentration), as used in this study, to provide a more comprehensive overview. For example, comparison across samples with the same cell densities can correct taxa that appears to be a ‘rare’ genus [46]. Alternatively, qPCR (with and without propidium monoazide/PMA treatment) together with heterotrophic plate counts (HPC) can be applied to quantify total and viable opportunistic bacteria of concern in full scale chlorinated DWDSs [62]. We also foresee the increased importance of metaproteomics and metabolomics [94,95] to provide a complementary understanding of drinking water microbial communities.

The bacteria spiking approach into tap water suspension applied in this study could be employed further to investigate the biological (in)stability of RO-treated water (or any membrane-treated water), e.g., potential growth assay (via model organism or through simulation of (wastewater) contamination. Evaluation of the possible influences from water constituents (organic or inorganic compounds) present in (but not limited to) chlorinated tap water towards DNA extraction yield can be done based on our membrane spiking approach.

## Conclusions

We evaluated the DNA recovery from chlorinated drinking water as a function of sampling volumes, presence and absence of a low-level residual chlorine (0.2 mg/L), and the effect of spiking a known *E*. *coli* concentration (into the water and onto the membrane filters). Samples with low bacterial cell content/density like RO-produced drinking water with residual chlorine had low DNA quantity that failed to be improved by higher filtration volumes and dechlorination. The DNA yield was below the minimum criteria for downstream (sequencing) molecular analysis. From the *E*. *coli* spiking experiments, we demonstrated that a low level of chlorine (0.2 mg/L) present in tap water had a destructive effect on the *E*. *coli* cells and their DNA, and likewise on the planktonic bacteria and any extracellular DNA present in a chlorinated drinking water network. The water spiking approach used in this study is advantageous for studying the biostability of high-quality drinking water. The membrane spiking is useful for evaluating effects from water matrix constituents towards DNA extraction yield. The findings from this study expand the sources of bias that may affect the interpretation of sequencing data that are intended for studying the microbial community structure and function in chlorinated drinking water systems.

## Supporting information

S1 Fig Gel electrophoresis image of the extracted (genomic DNA) of samples with different filtration volumes sample shows prominent absence of intact DNA (similar to low DNA integrity number (DIN) caused by degradation). DNA Integrity Number (DIN) is based on the molecular weight of intact DNA (low DIN: DNA with lower band position; high DIN: with higher band position). 1% Agarose gel (1X TAE buffer), 100V 25 mins, sample 1 μl loading, control 10 ng loading.
(DOCX)

S2 Fig A. Morphology of the membrane surface after filtration of MilliQ and original tap water before (A) and after dechlorination (B). The surface appearance of the membrane filter used for biomass filtration was observed at zero and three hours contact times after adding 10% (v/v) sodium metabisulfate (SMBS) (Na_2_S_2_O_5_) to quench the residual chlorine. B. Morphology of the membrane surface used for filtration of tap water containing residual chlorine (A) and dechlorinated (added with 10% SMBS) tap water (B) followed with *E*. *coli* spiking concentration of 10^7^ cells/cm^2^.
(DOCX)

S3 Fig Relationship between the expected and measured cell concentration of *E*. *coli* by flow cytometry plotted on log-log scale. A good linear correlation was observed between expected *E*. *coli* concentration and measured concentration by flow cytometry (FCM) from 10^3^ to 10^7^ cells/mL. Limit of detection of the FCM was as low as 1000 cells/mL in this study.
(DOCX)

S4 Fig Three dimensional fluorescence spectra of RO-treated tap water used in this study. A spectrofluorophotometer 3D-fluorescence excitation-emission matrix (3D-FEEM) was used to detect the fluorescence fingerprints of dissolved organic matters present in the tap water samples. Warm color panel indicates higher while cold color indicates lower abundance abundance of a particular organic fingerprints.
(DOCX)

S1 Table Characteristics of tap water used in the batch experiments, means ± SD. (DOCX)

S2 Table Details of *Escherichia coli* spiking experiment and cell recovery on tap water suspension and membrane (without and after filtration of tap water). Mean values (± SD) are shown for each variable (except cell recovery) based on triplicate samples. The effective membrane filter (0.2 μm, 47 mm diameter sterile mixed ester cellulose membrane) area used for tap water filtration was 11.58 cm^2^.
(DOCX)

S3 Table Organics and inorganics composition of tap water samples. (DOCX)

S1 Text Detection limit of flow cytometry used in this study-methods. (DOCX)

S2 Text Scanning Electron Microscopy (SEM) analysis-methods. (DOCX)

S3 Text PCR amplification of extracted DNA-methods. (DOCX)
