# Supplementary material for: Evaluation of DNA extraction yield from a chlorinated drinking water distribution system
Source: PLoS One. 2021 Jun 24;16(6):e0253799. doi: 10.1371/journal.pone.0253799 (PMC8224906; doi:10.1371/journal.pone.0253799)
Supplement: S1 Fig — DNA Integrity Number (DIN) is based on the molecular weight of intact DNA (low DIN: DNA with lower band position; high DIN: with higher band position). 1% Agarose gel (1X TAE buffer), 100V 25 mins, sample 1 μl loading, control 10 ng loading. (DOCX) [file pone.0253799.s001.docx]

**
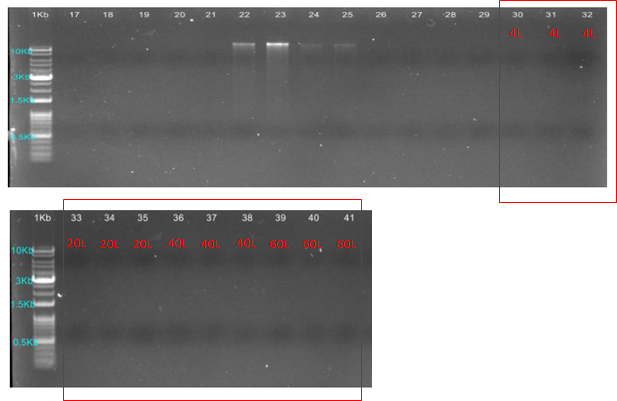
**

**S1 Fig. Gel electrophoresis image of the extracted (genomic DNA) of samples with different filtration volumes sample shows prominent absence of intact DNA (similar to low DNA integrity number (DIN) caused by degradation).** DNA Integrity Number (DIN) is based on the molecular weight of intact DNA (low DIN: DNA with lower band position; high DIN: with higher band position) [1]. 1% Agarose gel (1X TAE buffer), 100V 25 mins, sample 1 µl loading, control 10 ng loading.
