# Supplementary material for: Evaluation of DNA extraction yield from a chlorinated drinking water distribution system
Source: PLoS One. 2021 Jun 24;16(6):e0253799. doi: 10.1371/journal.pone.0253799 (PMC8224906; doi:10.1371/journal.pone.0253799)
Supplement: S2 Fig — A. Morphology of the membrane surface after filtration of MilliQ and original tap water before (A) and after dechlorination (B). The surface appearance of the membrane filter used for biomass filtration was observed at zero and three hours contact times after adding 10% (v/v) sodium metabisulfate (SMBS) (Na2S2O5) to quench the residual chlorine. B. Morphology of the membrane surface used for filtration of tap water containing residual chlorine (A) and dechlorinated (added with 10% SMBS) tap water (B) followed with E. coli spiking concentration of 107 cells/cm2. (DOCX) [file pone.0253799.s002.docx]

**
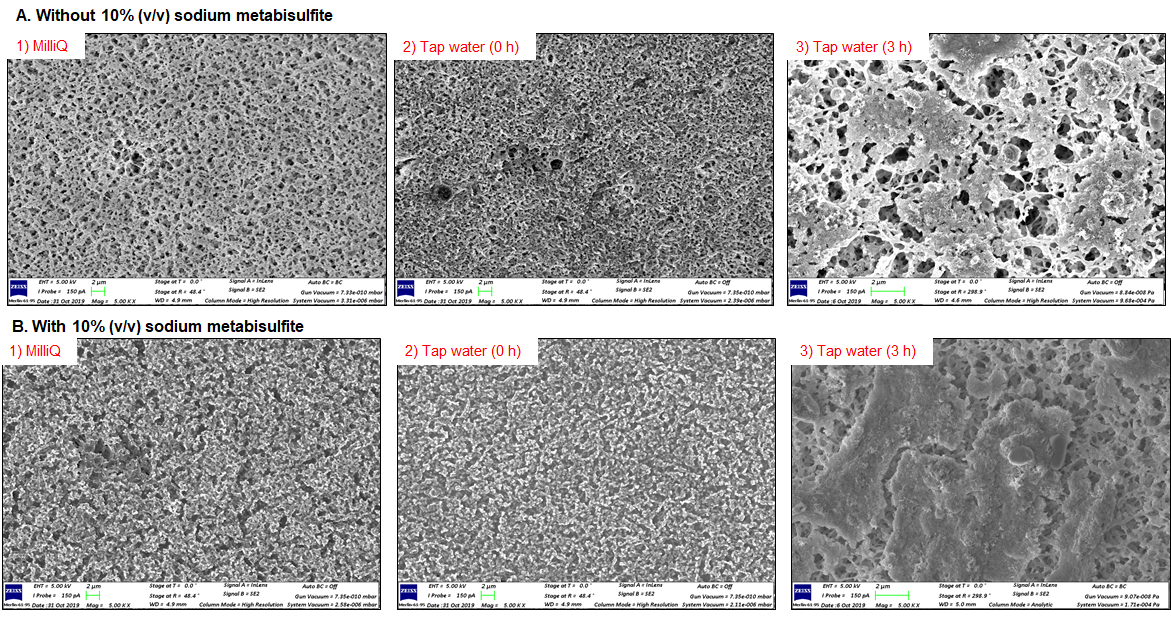
**

**S2A Fig. Morphology of the membrane surface after filtration of MilliQ and original tap water before (A) and after dechlorination (B).** The surface appearance of the membrane filter used for biomass filtration was observed at zero and three hours contact times after adding 10% (v/v) sodium metabisulfate (SMBS) (Na_2_S_2_O_5_) to quench the residual chlorine.

**
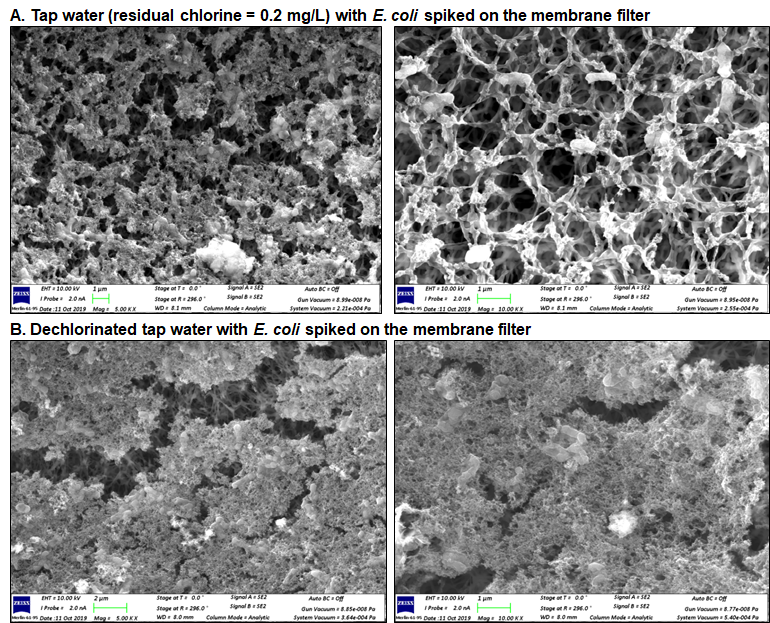
**

**S2B Fig. Morphology of the membrane surface used for filtration of tap water containing residual chlorine (A) and dechlorinated (added with 10% SMBS) tap water (B) followed with *E. coli* spiking concentration of 10^7^ cells/cm^2^.**

s
