# Supplementary material for: Evaluation of DNA extraction yield from a chlorinated drinking water distribution system
Source: PLoS One. 2021 Jun 24;16(6):e0253799. doi: 10.1371/journal.pone.0253799 (PMC8224906; doi:10.1371/journal.pone.0253799)
Supplement: S3 Fig — A good linear correlation was observed between expected E. coli concentration and measured concentration by flow cytometry (FCM) from 103 to 107 cells/mL. Limit of detection of the FCM was as low as 1000 cells/mL in this study. (DOCX) [file pone.0253799.s003.docx]

**
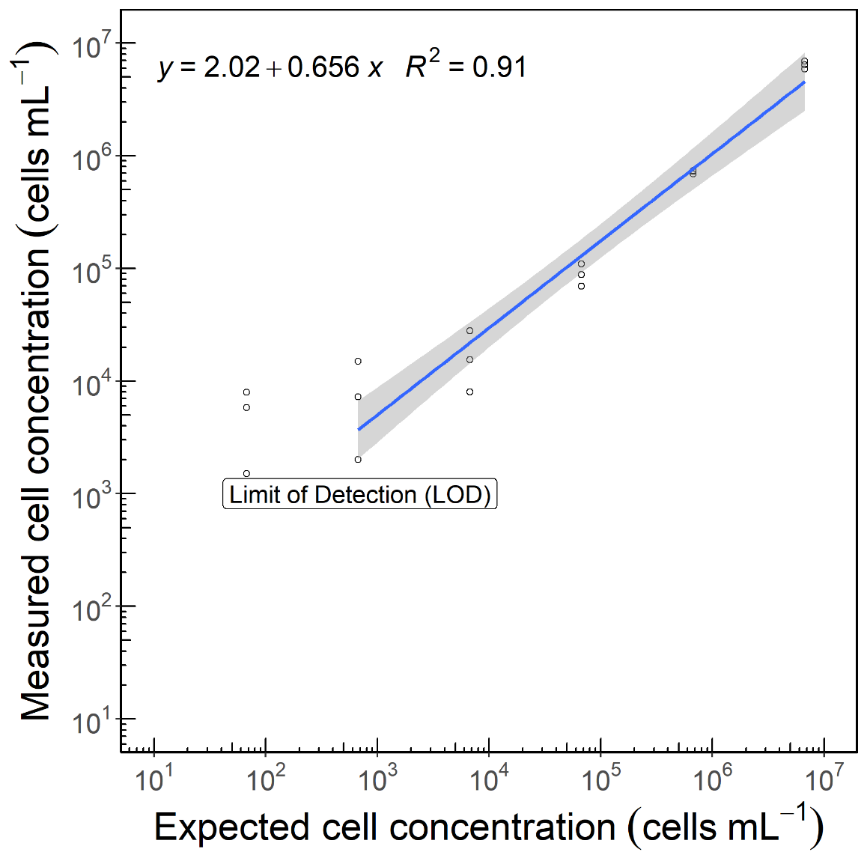
**

**S3 Fig. Relationship between the expected and measured cell concentration of *E. coli* by flow cytometry plotted on log-log scale.** A good linear correlation was observed between expected *E. coli* concentration and measured concentration by flow cytometry (FCM) from 10^3^ to 10^7^ cells/mL. Limit of detection of the FCM was as low as 1000 cells/mL in this study.
