# Supplementary material for: Evaluation of DNA extraction yield from a chlorinated drinking water distribution system
Source: PLoS One. 2021 Jun 24;16(6):e0253799. doi: 10.1371/journal.pone.0253799 (PMC8224906; doi:10.1371/journal.pone.0253799)
Supplement: S4 Fig — A spectrofluorophotometer 3D-fluorescence excitation-emission matrix (3D-FEEM) was used to detect the fluorescence fingerprints of dissolved organic matters present in the tap water samples. Warm color panel indicates higher while cold color indicates lower abundance abundance of a particular organic fingerprints. (DOCX) [file pone.0253799.s004.docx]

**

**

**S4 Fig. Three dimensional fluorescence spectra of RO-treated tap water used in this study.** A spectrofluorophotometer 3D-fluorescence excitation-emission matrix (3D-FEEM) was used to detect the fluorescence fingerprints of dissolved organic matters present in the tap water samples. Warm color panel indicates higher while cold color indicates lower abundance of a particular organic fingerprints.
