# Supplementary material for: Evaluation of DNA extraction yield from a chlorinated drinking water distribution system
Source: PLoS One. 2021 Jun 24;16(6):e0253799. doi: 10.1371/journal.pone.0253799 (PMC8224906; doi:10.1371/journal.pone.0253799)
Supplement: S1 Table — (DOCX) [file pone.0253799.s005.docx]

**S1 Table.** **Characteristics of tap water used in the batch experiments,** means (± SD). Spiking was done by adding inoculum of *E. coli* to reach the total concentration of 10^8^ cells in a total four liter volume. n.d means not detected (total chlorine ≤ 0.01 mg/L).

|  | | **Chlorinated tap water (T)** | | | **Dechlorinated tap water (TD)** | |
| --- | --- | --- | --- | --- | --- | --- |
|  | **pH** | | **Total Chlorine (mg/L)** | **pH** | | **Total Chlorine (mg/L)** |
| **Without *E. coli* spiking** | 9.16 (± 0.03) | | 0.20 (± 0.01) | 8.98 (± 0.03) | | n.d (≤ 0.01) |
| **Sterile water +*E. coli* spiking** | 7.60 (± 0.04) | | 0.21 (± 0.01) | 7.56 (± 0.02) | | n.d (≤ 0.01) |
| **Non-sterile water + *E. coli* spiking** | 7.66 (± 0.03) | | 0.20 (± 0.01) | 7.71 (± 0.02) | | n.d (≤ 0.01) |
