# Supplementary material for: Evaluation of DNA extraction yield from a chlorinated drinking water distribution system
Source: PLoS One. 2021 Jun 24;16(6):e0253799. doi: 10.1371/journal.pone.0253799 (PMC8224906; doi:10.1371/journal.pone.0253799)
Supplement: S2 Table — Mean values (± SD) are shown for each variable (except cell recovery) based on triplicate samples. The effective membrane filter (0.2 μm, 47 mm diameter sterile mixed ester cellulose membrane) area used for tap water filtration was 11.58 cm2. (DOCX) [file pone.0253799.s006.docx]

**S2 Table. Details of *Escherichia coli* spiking experiment and cell recovery on tap water suspension and membrane (without and after filtration of tap water).** Mean values (± SD) are shown for each variable (except cell recovery) based on triplicate samples. The effective membrane filter (0.2 µm, 47 mm diameter sterile mixed ester cellulose membrane) area used for tap water filtration was 11.58 cm^2^.

|  | **Initial cell count before spiking (cells/mL)** | ***E. coli* cell dosage used for spiking (cells)** | **Final cell count after spiking (cells/mL)** | **Total cells in 4 L batch volume (cells)^a^ or effective membrane filter area (cells)^b^** | **Cell recovery^c^ (%)** |
| --- | --- | --- | --- | --- | --- |
| **Spiking – tap water suspension** |  |  |  |  |  |
| **T sterile-EC** (tap—res. chlorine sterile + *E. coli*) | sterile | 4.5 × 10^8^ | 297 | 1.19 × 10^6^ | 0.2 |
| **T non-sterile-EC** (tap—res. chlorine non-sterile + *E. coli*) | 247 (± 35) |  | 247 | 9.87 × 10^5^ | 0.2 |
| **TD sterile-EC** (tap—dechlorinated sterile + *E. coli* | sterile |  | 1.09 × 10^5^ | 4.36 × 10^8^ | 96 |
| **TD** **non-sterile-EC** (tap—dechlorinated non-sterile + *E. coli*) | 247 (± 35) |  | 1.11 × 10^5^ | 4.44 × 10^8^ | 96 |
| **Spiking – membrane** |  |  |  |  |  |
| **-T+M+EC** (membrane + *E. coli* without water filtration) | Not applicable | 2.95 × 10^8^ | 2.36 × 10^7^ | 2.36 × 10^8^ | 80 |
| **+T+M+EC** (Membrane + *E. coli* after filtration of tap water—res.chlorine) | 247 (± 35) |  | 2.86 × 10^7^ | 2.86 × 10^8^ | 96 |
| **+TD+M+EC** (Membrane + *E. coli* after filtration of dechlorinated tap water) | 330 (± 125) |  | 2.92 × 10^7^ | 2.92 × 10^8^ | 99 |

**^a^**Cell concentration of *E. coli* in tap water (cells/mL). Total cells is the mean value calculated from the theoritical final cell count after *E. coli* spiking in the four liter volume of tap water prior filtration.

**^b^** Cell concentration in membrane concentrate in 10 mL sterile PBS (cells/mL) after spiking. Total cells is the mean value calculated after *E. coli* spiking onto the membrane (without and after filtration of tap water).

**^c^**Cell recovery is calculated from total cells obtained per expected (theoretical) cell count.
