# Supplementary material for: Evaluation of DNA extraction yield from a chlorinated drinking water distribution system
Source: PLoS One. 2021 Jun 24;16(6):e0253799. doi: 10.1371/journal.pone.0253799 (PMC8224906; doi:10.1371/journal.pone.0253799)
Supplement: S3 Table — (DOCX) [file pone.0253799.s007.docx]

**S3 Table. Organics and inorganics composition of tap water samples.**

| **Organics^a^** |  |
| --- | --- |
| Total organic carbon/TOC (mg/L) | 1.2 |
| **Inorganics^b^** |  |
| **Analyte** | **Concentration (µg/L)** |
| Sodium (Na) | 28600 |
| Calcium (Ca) | 3600 |
| Potassium (K) | 1930 |
| Magnesium (Mg) | 858.6 |
| Iron (Fe) | 124.7 |
| Zinc (Zn) | 74.4 |
| Alumunium (Al) | 17.3 |
| Strontium (Sr) | 9.0 |
| Nickel (Ni) | 4.8 |
| Lead (Pb) | 2.5 |
| Gold (Au) | 1.7 |
| Copper (Cu) | 1.7 |
| Palladium (Pd) | 1.2 |
| Lithium (Li) | 1.1 |
| Chromium (Cr) | ≤ 1 |
| Cobalt (Co) | N.D |
| Silver (Ag) | ≤ 1 |
| Mercury (Hg) | ≤ 1 |
| Scandium (Sc) | ≤ 1 |
| Manganese (Mn) | ≤ 1 |
| Barium (Ba) | ≤ 1 |
| Vanadium (V) | ≤ 1 |
| Titanium (Ti) | ≤ 1 |
| Beryllium (Be) | ≤ 1 |
| Cadmium (Cd) | ≤ 1 |

**^a^**Total organic carbon concentration was determined by TOC analyzer (Shimadzu, USA).

**^b^**Inductively coupled plasma-mass spectrometry (ICP-MS, Agilent USA) was used to determine trace elements (inorganics) in the tap water sample. The lowest detection limit of this machine was 1 µg/L.

N.D means no detection.
