# Supplementary material for: Evaluation of DNA extraction yield from a chlorinated drinking water distribution system
Source: PLoS One. 2021 Jun 24;16(6):e0253799. doi: 10.1371/journal.pone.0253799 (PMC8224906; doi:10.1371/journal.pone.0253799)
Supplement: S1 Text — (DOCX) [file pone.0253799.s008.docx]

**S1 Text. Detection limit of flow cytometry used in this study-methods.**

To estimate the lower detection limit of flow cytometry (FCM) used in this study, a dilution series was made consisting of *Escherichia coli* cells in a sterile PBS solution (1x concentration). *E. coli* cells were grown from pure culture as described previously (see *Preparation of E. coli cell stock solution*-materials and methods section-). Fresh 10-fold dilution series was made with concentrations ranging from ~10^8^ to 10^1^ cells/mL. The measurement was done in triplicates using FCM with the same setting and procedure described (see *Quantification of bacterial cell concentration using flow cytometry*-materials and methods section). The results were plotted on a log-log scale.
