# Supplementary material for: Evaluation of DNA extraction yield from a chlorinated drinking water distribution system
Source: PLoS One. 2021 Jun 24;16(6):e0253799. doi: 10.1371/journal.pone.0253799 (PMC8224906; doi:10.1371/journal.pone.0253799)
Supplement: S2 Text — (DOCX) [file pone.0253799.s009.docx]

**S2 Text. Scanning Electron Microscopy (SEM) analysis-methods.**

To investigate and visualize the morphology of the membrane used for water filtration, SEM analysis was conducted following the method described by Schneider et al. (2005) [2]. In brief, a small part of the membrane was cut from the middle part using sterile tweezers. The sections were fixed in 2% (v/v) glutaraldehyde and 4% (v/v) formaldehyde in distilled water for 2 h. The samples were immersed in a series of diluted ethanol with concentration 50%, 70% and 90% (v/v) for 10 min each and then rinsed three times in 100% (v/v) ethanol. Samples were dried in a vacuum desiccator, metallized and analyzed with a Zeiss Merlin scanning electron microscopy (Zeiss, Germany) provided by King Abdullah University of Science and Technology (KAUST) Core Labs.
