# Supplementary material for: Evaluation of DNA extraction yield from a chlorinated drinking water distribution system
Source: PLoS One. 2021 Jun 24;16(6):e0253799. doi: 10.1371/journal.pone.0253799 (PMC8224906; doi:10.1371/journal.pone.0253799)
Supplement: S3 Text — (DOCX) [file pone.0253799.s010.docx]

**S3 Text. PCR amplification of extracted DNA-methods.**

The extracted DNA from the different filtration volumes of dechlorinated tap water was amplified via polymerase chain reaction (PCR) using the TaqPCR Master Mix kit (Qiagen, Germany). The primer pairs used were specific for conserved 16s rRNA gene sequences of bacterial domain: 27F (AGAGTTTGATCMTGGCTCAG) and 1492R (TACGGYTACCTTGTTACGACTT) [3]. For each sampling volume (4, 20, 40, and 60 L), PCR was conducted in three independent replicates using the following conditions: i) initial denaturation at 95ºC for 6 minutes; followed by 30 cycles of ii) denaturation at 94ºC for 1 minute, iii) annealing at 58ºC for 60 seconds, iv) extension at 72ºC for 90 seconds; and ended with v) final extension at 72ºC for 5 minutes. Each PCR reaction consists of 10 µL GoTaq PCR mix, 2 µL (2 µM) of the forward primer, 2 µL (2 µM) of the reverse primer, 1 µL nuclease-free water, and 5 µL of template DNA with an addition of one negative control containing nuclease-free water instead of DNA template. PCR products were quantified with Qubit^®^ 4 Fluorometer (Thermo Fisher Scientific, USA) and Qubit double-stranded DNA (dsDNA) high-sensitivity (HS) Assay Kit (range 0.01 - 100 ng/µL) (Thermo Fisher Scientific, USA).

**References**

1. Manzari C, Oranger A, Fosso B, Piancone E, Pesole G, D'Erchia AM. Accurate quantification of bacterial abundance in metagenomic DNAs accounting for variable DNA integrity levels. Microb Genom. 2020. Epub 2020/08/05. doi: 10.1099/mgen.0.000417. PubMed PMID: 32749951.

2. Schneider R, Ferreira L, Binder P, Ramos J. Analysis of foulant layer in all elements of an RO train. Journal of Membrane Science. 2005;261(1-2):152-62. doi: 10.1016/j.memsci.2005.03.044.

3. Fredriksson NJ, Hermansson M, Wilén B-M. The Choice of PCR Primers Has Great Impact on Assessments of Bacterial Community Diversity and Dynamics in a Wastewater Treatment Plant. PLOS ONE. 2013;8(10):e76431. doi: 10.1371/journal.pone.0076431.
